# Investigation of MnO_2_ and Ordered Mesoporous Carbon Composites as Electrocatalysts for Li-O_2_ Battery Applications

**DOI:** 10.3390/nano6010021

**Published:** 2016-01-18

**Authors:** Chih-Chun Chin, Hong-Kai Yang, Jenn-Shing Chen

**Affiliations:** Department of Applied Chemistry, National University of Kaohsiung, Kaohsiung City 811, Taiwan; ccchin17@gmail.com (C.-C.C.); hong-kai84@hotmail.com (H.-K.Y.)

**Keywords:** MnO_2_/C, cathode, lithium-oxygen battery, rotating ring-disk electrode

## Abstract

The electrocatalytic activities of the MnO_2_/C composites are examined in Li-O_2_ cells as the cathode catalysts. Hierarchically mesoporous carbon-supported manganese oxide (MnO_2_/C) composites are prepared using a combination of soft template and hydrothermal methods. The composites are characterized by X-ray powder diffraction, scanning electron microscopy, transmission electron microscopy, small angle X-ray scattering, The Brunauer–Emmett–Teller (BET) measurements, galvanostatic charge-discharge methods, and rotating ring-disk electrode (RRDE) measurements. The electrochemical tests indicate that the MnO_2_/C composites have excellent catalytic activity towards oxygen reduction reactions (ORRs) due to the larger surface area of ordered mesoporous carbon and higher catalytic activity of MnO_2_. The O_2_ solubility, diffusion rates of O_2_ and O_2_^•−^ coefficients ($D_{O_{2}}$ and $D_{O_{2}^{•-}}$), the rate constant (*k_f_*) for producing O_2_^•−^, and the propylene carbonate (PC)-electrolyte decomposition rate constant (*k*) of the MnO_2_/C material were measured by RRDE experiments in the 0.1 M TBAPF_6_/PC electrolyte. The values of *k_f_* and *k* for MnO_2_/C are 4.29 × 10^−2^ cm·s^−1^ and 2.6 s^−1^, respectively. The results indicate that the MnO_2_/C cathode catalyst has higher electrocatalytic activity for the first step of ORR to produce O_2_^•−^ and achieves a faster PC-electrolyte decomposition rate.

## 1. Introduction

Energy storage devices with high energy and power densities are being developed for use as power sources for electric vehicles (EV) and hybrid electric vehicles (HEV) [1,2,3]. Over the past few decades, the vast majority of relevant research has focused on upgrading the performance of conventional lithium-ion batteries for EV or HEV applications; however, their energy densities and specific charge capacities still fail to satisfy commercial requirements such as long-range driving, low cost, and fast charging [1,2,4]. In recent years, rechargeable nonaqueous Li-air batteries have attracted much interest owing to their low cost, environmental friendliness, and high theoretical energy density (~3500 Wh·kg^−1^), which is nearly equivalent to a nine-fold increase over conventional Li-ion batteries (~400 Wh·kg^−1^) [4,5,6,7]. Despite these favorable characteristics, their practical applications are still hampered by several serious challenges including limited rate capability, poor cycling stability due to the instability of the electrode and electrolyte, and low round-trip efficiency induced by the rather large polarization, resulting in a wide charge–discharge voltage gap [3,8,9,10,11,12,13,14,15]. These critical problems are highly attributable to the O_2_ cathode.

A typical rechargeable Li-O_2_ battery is constituted by a porous oxygen diffusion cathode, a lithium metal anode, and an Li^+^-conducting electrolyte. In general, the O_2_ cathode is an oxygen catalyst loaded with porous carbon material, which enables both Li_2_O_2_ deposition (oxygen reduction reactions, ORRs) and decomposition (oxygen evolution reactions, OERs) reactions to occur upon battery discharge and charge, respectively. Many reports [1,4,5,6,8,9,11,15,16,17,18] have pointed out that the electrochemical performance of Li-O_2_ batteries depends on many factors such as: the nature and microstructure of the O_2_ electrode, electrolyte formula (especially, the composition of solvent), O_2_ partial pressure, possible presence of reactive contaminants (e.g., trace water), and the choice of catalysts. In order to enhance the properties of rechargeable Li-O_2_ batteries, several strategies have been followed over the years to explore the electrolyte formula, choice, and microstructure design of the O_2_ electrode and optimization of the operating parameters [1,3,5,8,9,10,11].

Carbon materials with various nanostructures have been developed and used as O_2_ cathodes in Li-O_2_ batteries [4,6,10,19]. It has been well demonstrated that the performance of Li-O_2_ batteries is related to the properties of carbon, such as the morphology, surface area, porous structure, and conductivity [6,9,20]. The design of porous carbon cathodes requires larger intraparticulate voids and open frameworks in their architecture structure to accommodate the insoluble discharge products. These voids and frameworks should help improve discharge capacity and cycling performance [19,20,21]. Obviously, designing an optimum pore structure for carbon materials can effectively improve the electrochemical performance of Li-O_2_ batteries. Although various porous carbon structures have been explored, some studies have demonstrated that hierarchically porous honeycomb-like carbon cathodes with mesoporous/macroporous pore size can increase the specific capacity of Li-O_2_ batteries [4,5,6,15,19,20,21,22,23,24,25,26]. Moreover, it is well known that an ideal cathode catalyst can facilitate the complete reversibility of ORRs and OERs with low polarization in Li-O_2_ batteries [21]. Several potential catalysts have recently been proposed to promote ORRs and OERs, including nitrogen-doped carbon, metal oxides, metal nitrides, precious and nonprecious metals, *etc.* [1,3,8,13,15,19,27,28,29]. Among metal oxides, MnO_2_ is a catalyst material of great interest because of its low cost, environmental friendliness, abundance, and electrocatalytic activity for ORRs in Li-O_2_ batteries [13,28,30,31,32]. This study of Li-O_2_ batteries focuses on MnO_2_-based catalysts.

In the first part of this work, we created a hierarchically mesoporous carbon-supported β-manganese oxide (MnO_2_/C) as an O_2_ cathode material. We present a detailed study of the Li-O_2_ electrochemistry of the MnO_2_/C material using an electrolyte of 1 M LiPF_6_ in a propylene carbonate (PC, which was used in many of the initial works on Li-O_2_ batteries) solvent. Although there have been many studies of MnO_2_/C materials for Li-O_2_ battery applications, few studies have examined the poor stability of the electrolyte due to its reaction with the superoxide radical (O_2_^•−^) produced upon the discharge at the MnO_2_/C electrode. In this work, the stability of the electrolyte against the O_2_^•−^ of the MnO_2_/C electrode was first explored by the RRDE technique. The RRDE was developed about 50 years ago and has been verified to be a powerful tool for the study of electrochemical reactions. RRDE consists of two concentric electrodes (disk and ring electrodes) in a cylindrical holder with both of the electrodes facing downward into the solution. Products generated at the disk reaction are swept outward by the convection caused by rotation, and can be detected electrochemically at the ring by fixing the potential on the ring electrode. In this study, a disk electrode coated with MnO_2_/C materials and a Pt ring electrode was fixed at an O_2_^•−^/O_2_ oxidation potential to collect the O_2_^•−^ ions in electrolytes. Therefore, in the second part, we emphasize aspects of the PC-based electrolyte reaction against O_2_^•−^ and the related kinetic information of O_2_^•−^ in the MnO_2_/C electrode by studying rotating ring disk electrode (RRDE) experiments and using a lithium-free non-aqueous electrolyte due to the stability of the intermediate O_2_^•−^. In addition, the oxygen solubility in the electrolyte and the oxygen diffusion velocity throughout the whole O_2_ electrode play key roles in determining battery performance, especially at high current densities [33]. In this work, the O_2_ solubility, diffusion rates of O_2_ and superoxide radical (O_2_^•−^) coefficients ($D_{O_{2}}$ and $D_{O_{2}^{•-}}$), rate constant (*k_f_*) for producing O_2_^•−^, and PC-electrolyte decomposition rate constant (*k*) of the MnO_2_/C electrode were quantified.

## 2. Experimental Methods

MnO_2_/C composites were prepared by supramolecular self-assembly methods followed by a hydrothermal process. A modification of the mesoporous metal oxides and carbon nanocomposites procedure of Huang *et al.* [34] was applied to synthesize the MnO_2_/C composites. The first step was to synthesize a 20 wt. % resol ethanolic solution according to an established method [34,35]. A solution was prepared by dissolving 1.5 g of triblock copolymer Pluronic F127 (OH(CH_2_CH_2_O)*_n_*-(CH_2_CH(CH_3_)O)*_m_*-(CH_2_CH_2_O)*_n_*H, EO_106_PO_70_EO_106_, Sigma Aldrich, St. Louis, MO, USA) in 10 g of anhydrous ethanol, then 5 g 20 wt. % resol ethanolic solution and 0.28 g MnCl_2_·4H_2_O (J.T. Baker, 99.8%) were added into the above solution slowly under stirring for 30 min at an ambient temperature. The homogeneous mixture was then transferred into a Petri dish at an ambient temperature for 24 h. After being dried, the films were heated at 100 °C for another 24 h to form orange transparent membranes. The as-made products were scraped from the Petri dish and ground into powders and then calcinated at 400 °C for 5 h under an Ar atmosphere with a heating rate of 1 °C·min^−1^ to yield Mn/C powders. To obtain MnO_2_/C composites, the as-prepared Mn/C powders were subjected to a hydrothermal process at 180 °C for 12 h with 0.22 g KMnO_4_ (J.T. Baker) and 30 mL of deionized water in a Teflon-lined stainless steel autoclave.

A Rigaku-D/MaX-2550 diffractometer (Rigaku, Tokyo, Japan) with Cu K_α_ radiation (λ = 1.54 Å) was used to obtain X-ray diffraction (XRD) patterns for the samples. Small angle X-ray scattering (SAXS) measurements were taken on a Nanostar U small-angle X-ray scattering system (Bruker, Karlsruhe, Germany) using Cu K_α_ radiation (40 kV, 35 mA). The morphology of the sample was observed using a scanning electron microscope (SEM, Hitachi S-3400 (Hitachi Limited, Tokyo, Japan)) and transmission electron microscope (TEM, JEOL JEM-3010 (JEOL, Tokyo, Japan)). Selected area electron diffraction (SAED) was applied to examine samples’ crystallinity. The Brunauer–Emmett–Teller (BET) method was used to measure the specific surface area of the powders (ASAP2020). The residual carbon content of the samples was measured by an automatic elemental analyzer (EA, Elementar vario, EL III (Elementar Analysensysteme GmbH, Hanau, Germany)).

For electrochemical evaluation, the MnO_2_/C electrodes were prepared by wet coating, and were made from as-prepared MnO_2_/C composites with super P and a poly(vinylidene difluoride) (PVDF) binder (MKB-212C, Atofina, Serquigny, France) in a weight ratio of 64:16:20. The MnO_2_/C composites and super-P were first added to a solution of PVDF in *N*-methyl-2-pyrrolidone (NMP, Riedel-deHaen, Seelze, Germany). To make a slurry with an appropriate viscosity, the mixture was stirred for 20 min at room temperature using a magnetic bar, and then for 5 min using a turbine at 2000 rpm. The resulting slurry was coated onto a piece of separator (Celgard 2400, Charlotte, NC, USA) and dried at 60 °C under vacuum for 12 h. The coating had a thickness of ~100 μm with an active material mass loading of 8 ± 1 mg·cm^−2^. The quantity of active materials on the electrodes was kept constant. Electrodes were dried overnight at 100 °C under a vacuum before being transferred into an argon-filled glove box for cell assembly. The Li-O_2_ test cell (EQ-STC-LI-AIR, MTI Corporation, Richmond, CA, USA) was constructed with lithium metal as the negative electrode and the MnO_2_/C electrode as the positive electrode. A solution of 1 M LiPF_6_ in a PC solvent was used as the electrolyte in all cells. After assembly, the test cell was taken away from the Ar-filled glove box and attached to a gas pipe that was constantly purged with dry O_2_. Electrochemical tests were carried out after the cell was flushed with O_2_ for 6 h. The cells were cycled galvanostatically with a BAT-750B (Acu Tech System, Taipei, Taiwan) at a constant current of 100 mA/g with a voltage region of 2.0–4.3 V *vs.* Li/Li^+^ at room temperature.

**Figure 1:**
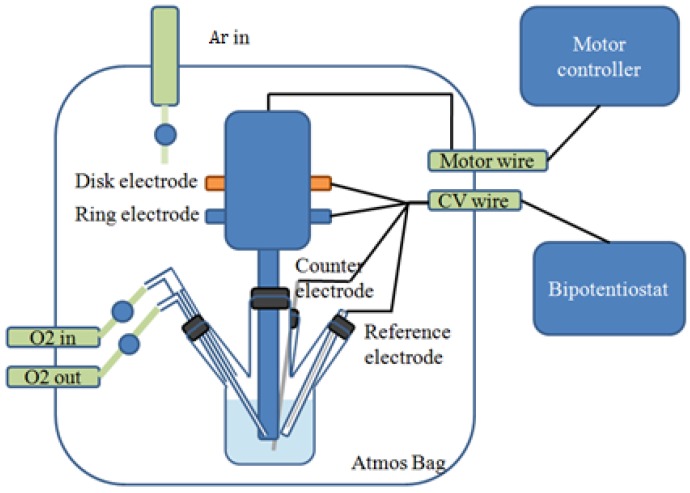
Schematic of a four-neck, jacketed glass cell with a rotating ring-disk electrode (RRDE) system.

For the RRDE experiments, the RRDE system (AFMT134DCPTT, Pine Research Instrumention, Durham, NC, USA) with interchangeable disk consisted of a 5 mm diameter glassy carbon electrode and a Pt ring electrode (1 mm width) with a 0.5 mm gap between them. The collection efficiency with this geometry is 0.24. The rotating ring-disk assembly was operated on a Pine AFMSRX rotator and CH705 Bipotentiostat (CH Instruments, Austin, TX, USA) with a computerized interface. Experiments were conducted using a three-electrode cell containing 10 mL of the electrolyte of interest and assembled in a dry Ar-filled AtmosBag (Sigma-Aldrich Z108450, St. Louis, MO, USA). Figure 1 shows the schematic of a four-neck, jacketed glass cell with the RRDE system. The counter electrode was a Li foil connected to a Ni wire, which was isolated by a layer of Celgard 2400 separator to prevent convective oxygen transport to the electrode. The Ag/Ag^+^ reference electrode consisted of an Ag wire immersed into 0.1 M AgNO_3_ in CH_3_CN and sealed with a vycor frit at its tip. All potentials in this study were referenced to the Li/Li^+^ potential scale (volts *vs.* Li^+^/Li or V_Li+_), obtained by calibration of the reference electrode against a fresh lithium wire before the experiments (0 V_Li_ = −3.46 ± 0.01 V *vs.* Ag/Ag^+^). The working electrode consisted of a catalyst-covered glassy carbon disk and was immersed into the Ar or O_2_-purged electrolyte for 30 min before each experiment. Prior to the RRDE measurements, Alternating current (AC) impedance measurements were carried out to determine the uncompensated ohmic electrolyte drop between working and reference electrodes by applying a 10 mV perturbation (0.1 MHz to 10 mHz) at the open circuit. IR (drop) correction to remove ohmic losses was performed by considering a total cell resistance of ~293 Ω measured by AC impedance. The capacitive-corrected ORR currents were calculated by subtracting the current measurement under Ar from that obtained in pure O_2_ under identical scan rates, rotation speeds, and catalyst loadings.

## 3. Results and Discussion

**Figure 2:**
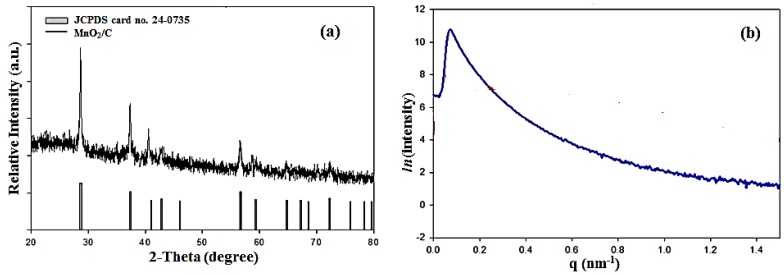
(**a**) Wide-angle X-ray diffraction (XRD) patterns; (**b**) small angle X-ray scattering (SAXS) patterns of MnO_2_/C composites.

The phase composition and structure of the prepared MnO_2_/C composites were examined by the wide-angle XRD and SAXS patterns given in Figure 2a,b. As shown in Figure 2a, all peaks can be identified as a pure and well-crystallized β-MnO_2_ phase (JCPDS 24-0735) with an ordered tetragonal structure indexed to the P42/mnm space group. Moreover, the XRD curves did not show any evidence of the formation of crystalline or amorphous carbon. It appears that when using resol/Pluronic F127 templates as a carbon source, the final product is most likely to remain amorphous or in a low crystalline carbon state. The appearance of the scattering peak in the SAXS pattern, as shown in Figure 2b, indicates the long-range regularity and highly ordered nature of the mesoporous structures of the prepared MnO_2_/C composite.

**Figure 3:**
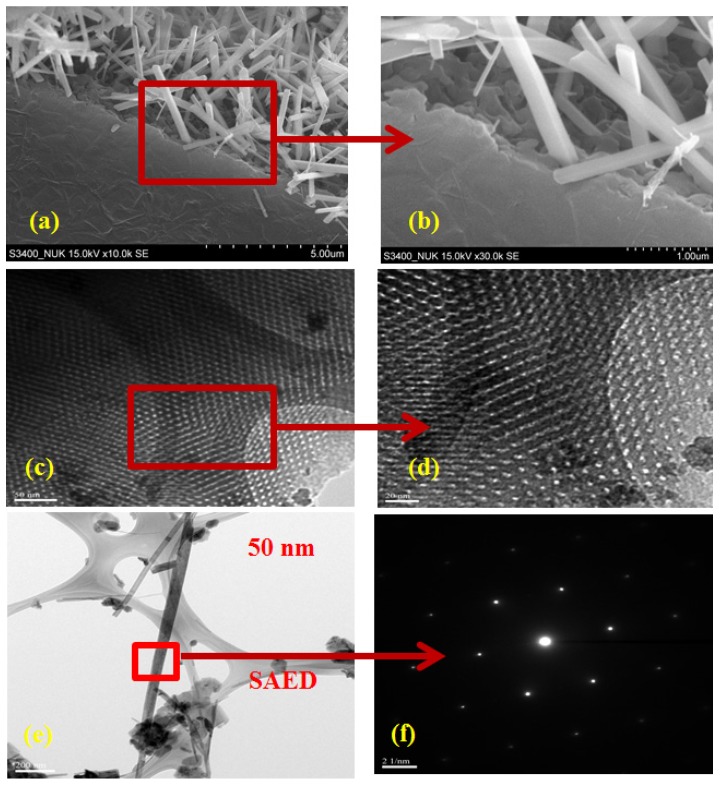
Scanning electron microscope (SEM) images (**a**) MnO_2_/C composites; (**b**) high magnification of the region marked with a square in (**a**); and transmission electron microscope (TEM) images (**c**,**e**) MnO_2_/C composites; (**d**) high magnification of the region marked with a square in (**c**); and (**f**) selected area electron diffraction (SAED) pattern of the region marked with a square in (**e**).

The morphology of the prepared MnO_2_/C composite was observed using SEM and TEM, as shown in Figure 3a–f. From the SEM images of the MnO_2_/C composite (Figure 3a,b), it is clear that the oriented tetragonal MnO_2_ nanorods are arranged on the surface of the carbon matrix. The prepared β-MnO_2_ nanorods, typically 2–3 μm in length, have a square cross-section with an edge length in the range of 200–300 nm. Figure 3c,d show the TEM images of the MnO_2_/C composite at different magnifications. Large domains of highly ordered stripe-like 1D channels are clearly observed. Figure 3e displays a TEM image of a typical nanorod with a smooth surface, and a SAED pattern based on a single nanorod (Figure 3f), indicating single-crystalline nature. The SEM and TEM analysis suggest that hollow MnO_2_ nanorods grow homogeneously on the ordered mesoporous carbon frameworks to form the structure of the hierarchically mesoporous MnO_2_/C composite.

**Figure 4:**
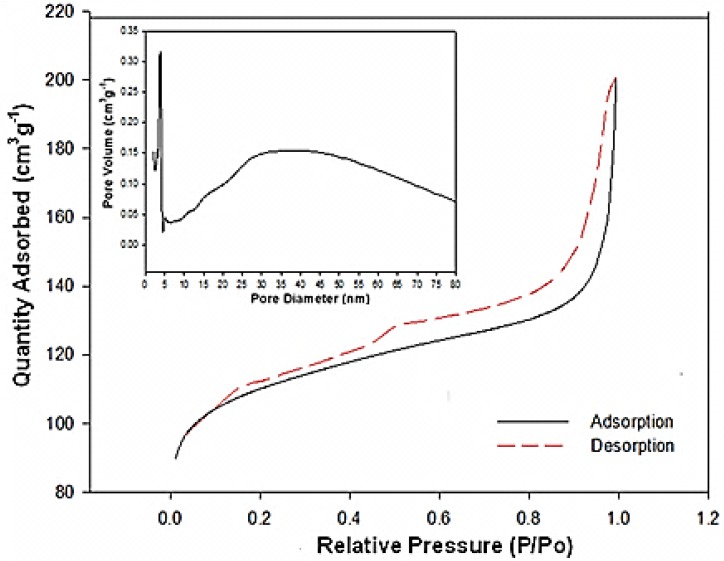
Nitrogen sorption isotherms of MnO_2_/C composites. The insert is the Barrett–Joyner–Halenda (BJH) desorption pore size distribution.

The pore structure of the mesoporous MnO_2_/C composite was determined by nitrogen adsorption-desorption isothermal measurements. As shown in Figure 4, the adsorption isothermal curve of the MnO_2_/C composite has a well-defined step as in typical IV classification with a H_1_-type hysteretic loop in the p/p_o_ range of 0.40–1.0, indicating mesoporous material character. These findings suggest that the MnO_2_/C composite sample does not contain framework-confined pores but is rather made up of individual nanorods. This is in agreement with the results from the SEM and TEM images. The Barrett–Joyner–Halenda (BJH) pore size distribution for the mesoporous MnO_2_/C composite, shown in the insert of Figure 4, reveals peaks centering at 4.8 and 35 nm. This result confirms that most of the pore channels in the ordered mesoporous carbon are not blocked by the loading of MnO_2_ nanorods. The nanoarchitecture of ordered mesoporous channels is maintained, which is desirable for the O_2_ electrode in Li-O_2_ batteries. Moreover, the measured BET surface area of the MnO_2_/C composite is relatively high, at about 424 m^2^·g^−1^. The hierarchical microstructure of the MnO_2_/C composite results in a large specific surface area. This is important for enhancing the electrochemical properties of an O_2_ cathode material.

**Figure 5:**
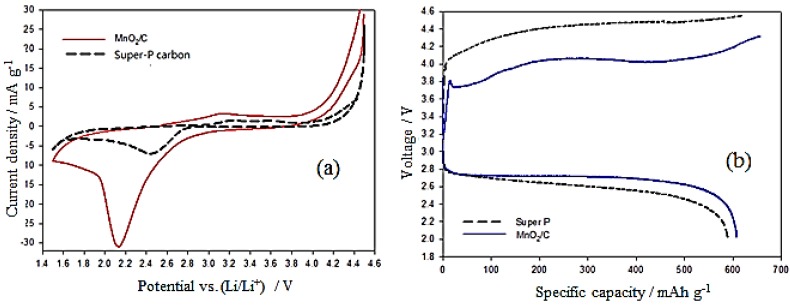
(**a**) CV curves were recorded at a scanning rate of 2 mV·s^−1^ for MnO_2_/C and Super-P carbon samples; (**b**) initial charge–discharge profiles for MnO_2_/C and Super P samples at a current density of 0.2 mA·cm^−2^.

MnO_2_ has been known as a highly active ORR catalyst for some time [30,32,36] and has recently been applied as an O_2_ cathode catalyst in Li-O_2_ batteries. Due to the studies of the electrocatalytic activity of MnO_2_, the following discussions regarding electrochemical tests make comparisons between Super-P carbon (SP) and MnO_2_/C materials. To better study the catalytic activity of the electrodes, cyclic voltammetry (CV) and charge-discharge voltage measurements were carried out. At first, CV was carried out in the Ar-purged electrolyte and subsequently in the same solution saturated with O_2_. The capacitively-corrected CV curves derived from both measurements are shown in Figure 5a. The CV plots of the O_2_ electrodes prepared from MnO_2_/C and SP cycled between 1.5 and 4.5 V with 2 mV·s^−1^ and the O_2_-saturated 1 M LiPF_6_/PC electrolyte are shown in Figure 5a. From the CV curves, the reduction peak voltage is shifted toward positive voltage, exhibiting electrocatalytic activity in the ORR of both samples. However, the MnO_2_/C offers more positive onset reduction peak potential and a larger peak current, which clearly indicate the superior electrocatalytic activity of MnO_2_/C compared to SP. Furthermore, the onset oxidation peaks appearing in the CV curves are about 2.7 and 2.9 V for MnO_2_/C and SP, respectively. This demonstrates that MnO_2_/C, with its lower onset oxidation peak, is more efficient for Li_2_O_2_ decomposition and has higher catalytic activity for the OER. The initial charge–discharge voltage profiles for both samples are shown in Figure 5b. The charge–discharge profiles of the MnO_2_/C electrode exhibit much lower charge overpotential than do those of the SP electrode, although the reduction of the total overpotential is only about 25%. The round-trip efficiencies of the Li-O_2_ batteries with a MnO_2_/C electrode were lower than those with the SP electrode. These results indicate that the MnO_2_/C composite can facilitate the complete reversibility of ORR and OER with low polarization for a Li-O_2_ battery. This finding is in good agreement with the CV measurement. The initial discharge capacities of the MnO_2_/C and SP electrodes were 612 mAh·g^−1^ and 589 mAh·g^−1^, respectively. The good electrochemical performance of the MnO_2_/C electrode may be due to the hierarchical mesostructure and large specific surface area, and the catalytic activity of the MnO_2_/C composite.

The rotating ring disk electrode (RRDE) technique was also used to investigate the kinetics of ORR since the ORR current is strongly relevant to hydrodynamic conditions [31]. Here, we used a glassy carbon (GC) electrode and an as-prepared MnO_2_/C composite coated on the GC (MnO_2_/C-GC) electrode as the working electrodes to study the stability of the electrolyte at the MnO_2_/C electrode. Many reports [1,5,9] have shown the O_2_^•−^ produced in the first step of the ORR upon battery discharge:

(1) $$O_{2}+e^{-}\overset{k_{f}}{\to }{O_{2}}^{•-}$$

The reaction between the O_2_^•−^ and the electrolyte is the critical problem that causes poor Li-O_2_ battery cyclability. In the PC-based electrolyte, the ethereal carbon atom in PC suffers from nucleophilic attacks by O_2_^•−^, yielding carbonate, acetate, and formate species (among others), according to Equation (2) [37,38]:

(2) $$PC+{O_{2}}^{•-}\overset{k}{\to }{{CO}_{3}}^{2-},HCOO^{-},CH_{3}COO^{-}$$

Here, we applied rotating disk electrode (RDE) voltammetry to measure the rate constant (*k_f_*) when reducing O_2_ to O_2_^•−^ for Equation (1) in the 0.1 M TBAPF_6_/PC electrolyte. The reaction rate constant, *k_f_*, can be evaluated via the Koutecky–Levich (K–L) equation for a first-order reaction as follows [31]:

(3) $$\frac{1}{i}=\frac{1}{i_{k}}+\frac{1}{i_{d}}$$

(4) $$i_{k}=nFk_{f}C_{O_{2}}$$

(5) $$i_{d}=0.62nF{D_{O_{2}}}^{2/3}\nu^{-1/6}C_{O_{2}}\omega^{1/2}$$

where *i_k_* and *i_d_* represent kinetics and diffusion limiting current density (A·m^−2^), respectively; *n* is the number of electrons exchanged in the electrochemical reaction; *F* is Faraday’s constant (96,485 C·mol^−1^); *k_f_* is the rate constant for Equation (1); $D_{O_{2}}$ is the diffusion coefficient of O_2_ in the solution; ν is the kinematic viscosity; ω is the angular frequency of the rotation; and $C_{O_{2}}$ is the saturation concentration of O_2_ in the solution. Additionally, knowing the values of ν and $D_{O_{2}}$ for an electrolyte, one can obtain the concentration of oxygen ($C_{O_{2}}$) and rate constant (*k_f_*) for Equation (1) by linearly fitting the K-L plots of *i*^−1^ *vs.* ω^−0.5^, as follows

(6) $$\frac{1}{i}=\frac{1}{i_{k}}+\frac{1}{0.62nFD_{O_{2}}^{2/3}\nu^{-1/6}C_{O_{2}}\omega^{1/2}}$$

**Figure 6:**
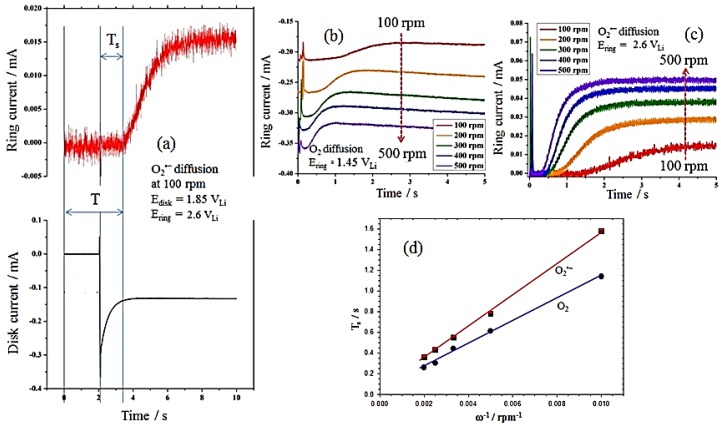
(**a**) Example of determination of the superoxide radical (O_2_^•−^) transit-time (*T_s_*) in O_2_-saturated solutions of 0.1 M TBAPF_6_ in propylene carbonate (PC) at ω = 100 rpm, E_disk_ = 1.85 V and E_ring_ = 2.6 V. Transit time (*Ts*) values at different rotation rates for the diffusion of (**b**) O_2_ and (**c**) O_2_^•−^; (**d**) relation between the inverse of the rotation speed and the transient time for O_2_ and O_2_^•−^.

**Figure 7:**
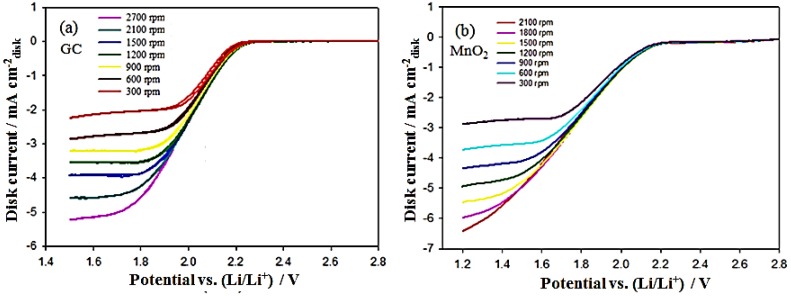
(**a**) Steady-state CV curves of a glassy carbon rotating disk electrode (RDE) in an O_2_-saturated 0.1 M TBAPF_6_/PC solution at a scan rate of 50 mV/s between 1.5 and 2.8 V_Li_ with different rotation rates. The insert is the Koutecky–Levich plot derived from the disc current values at 1.50 V_Li_; (**b**) steady-state CV curves of a MnO_2_/C RDE in an O_2_-saturated 0.1 M TBAPF_6_/PC solution at a scan rate of 50 mV/s between 1.2 and 2.8 V_Li_ with different rotation rates.

Prior to estimating the value of *k_f_* from the K–L equation, the kinematic viscosity (ν) of the electrolyte and the diffusion coefficients of O_2_ and O_2_^•−^ ($D_{O_{2}}$ and $D_{O_{2}^{•-}}$), need to be quantified. The value of ν for PC with 0.1 M TBAPF_6_ is 2.59 × 10^−2^ cm^2^·s^−1^ at 25 °C (ρ = 1.2 g·mL^−1^ and η = 3.13 mPa·s) and was measured by a Rheometer (Malvern Gemini, Malvern Instruments Ltd., Malvern, UK). For a known viscosity, the diffusion coefficients can be directly determined from the transit-time (*T_s_*) measurement by the RRDE technique, as reported previously [37,39]. Figure 6a shows an example of *T_s_* measurement in O_2_-saturated solutions of 0.1 M TBAPF_6_ in PC at ω = 100 rpm; *T_s_*, the origin of which is taken at time = 2 s (the time at which the disk is conducted cathodic potential at 1.85 V_Li_), is measured graphically from the intercept of the base steady ring current and the fast attenuate ring current line. Figure 6b,c show measurements of steady ring currents at the rotation rates (ω) of different electrodes, yielding *T_s_* values for O_2_ and O_2_^•−^. Then, the obtained *T_s_* is related to the ω and the ratio of ν and the diffusion coefficient (*D*), according to Equation (7) [37,39]:

(7) $$T_{s}=Κ{\left(\frac{\nu }{D}\right)}^{1/3}\omega^{-1}$$

where K is proportionally constant depending on the RRDE’s geometry; K = 43.1[log(*r*_2_/*r*_1_)]^2/3^ (for *T_s_*, reported in s and ω in rpm). For the RRDE used here, with *r*_1_ = 0.25 cm and *r*_2_ = 0.325 cm, the value of K is 10.1 rpm·s. Table 1 shows the estimated values of the diffusion coefficients of O_2_ and O_2_^•−^ calculated from Equation (7) based on the slopes of *T_s_ vs.* ω^−1^ obtained from Figure 6d. Figure 7a shows that well-defined O_2_ diffusion-limited currents are obtained for the ORR on a GC electrode in an O_2_-saturated 0.1 M TBAPF_6_/PC solution. The K–L plot for the disk current values at 1.50 V_Li_ reveals the expected linear relation between the inverse of the limiting current and ω^−0.5^ (see Equation (6)). As shown in Table 1, the concentration of oxygen ($C_{O_{2}}$) on the GC electrode was estimated from the slope of the K–L plot using the prior measured values of ν and $D_{O_{2}}$, where *n* = 1 (according to the reaction of Equation (1)). The value of $C_{O_{2}}$ is 6.1 M, which is higher than the finding of a previous report (4.8 M) [37]. This can be attributed to the larger O_2_ flow rate in this experiment. The estimated value of $C_{O_{2}}$ was also applied in the following calculations of the MnO_2_/C-GC electrode since the same operation parameters (*i.e.*, O_2_ flow rate, electrolyte composition, and amount) were used, as listed in Table 1. The rate constant for producing O_2_^•−^, *k_f_* for GC and the MnO_2_/C-GC electrodes can be obtained by linearly fitting the K–L plots of *i*^−1^ *vs.* ω^−0.5^ (see Equation (6)), as shown in Figure 7a,b. The values of *k_f_* for GC and the MnO_2_/C-GC electrodes are 1.92 × 10^−2^ cm·s^−1^ and 4.29 × 10^−2^ cm·s^−1^, respectively. This result indicates that the MnO_2_/C cathode catalyst exhibits a larger *k_f_* value, resulting from higher electrocatalytic activity for the first step of the ORR (see Equation (1)) which produces a higher concentration of O_2_^•−^.

**Table 1:** Summary of the electrolyte properties estimated with the proposed RRDE-based methodology and comparison with findings reported in the literature.

Disk Material/Electrolyte	ν (cm^2^·s^−1^)	$D_{O_{2}}$ (cm^2^·s^−1^)	$D_{O_{2}^{•-}}$ (cm^2^·s^−1^)	$C_{O_{2}}$ (mM)	Reference
GC/0.1 M TBAPF_6_, PC	2.6 × 10**^−^**^2^	1.9 × 10**^−^**^5^	8.6 × 10**^−^**^6^	6.1	This work
MnO_2_/C-GC/0.1 M TBAPF_6_, PC	2.6 × 10**^−^**^2^	1.9 × 10**^−^**^5^	1.8 × 10**^−^**^6^	6.1	This work
GC/0.2M TBATFSI, PC	2.6 × 10**^−^**^2^	2.5 × 10**^−^**^5^	6.8 × 10**^−^**^6^	4.8	[37]

**Figure 8:**
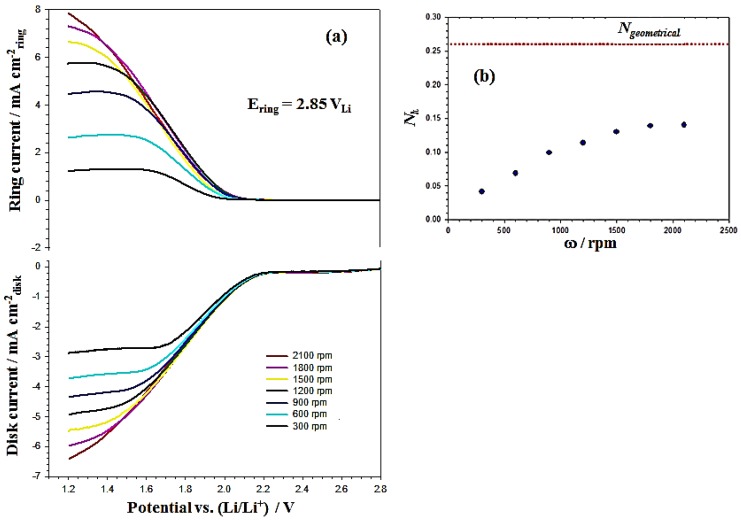
(**a**) RRDE profiles of MnO_2_/C recorded at 50 mV·s^−1^ in an O_2_-saturated 0.1 M TBAPF_6_/PC solution, at rotation rates between 300 and 2100 rpm with continuous holding of the Pt ring at 2.85 V_Li_; (**b**) evolution of the absolute ratio between the ring and disk current (*N_k_*) and the electrode rotation speed (ω).

Recently, Herranz *et al.* [37] used RRDE voltammetry to quantify the stability of an electrolyte against O_2_^•−^ by the rate constant (*k*) for Equation (2) According to their methods, the O_2_^•−^ produced at the disk electrode in Equation (1) and the amount of O_2_^•−^ were quantified at the ring electrode. The amount of O_2_^•−^ consumed depends on the effective transient time, *T_s_*, between the disk and the ring and the rate constant, *k*, for Equation (2). Longer *T_s_* and larger *k* values cause increasing consumption of O_2_^•−^ due to its reaction with the electrolyte, resulting in a lower O_2_^•−^ oxidation current at the ring. Therefore, the collection efficiency, *N_k_*, for O_2_^•−^ at the ring electrode decreases with increasing transient time, which, in turn, depends on the geometry of ring and disk electrode, the diffusion coefficient of O_2_^•−^ in the electrolyte, $D_{O_{2}^{•-}}$, and the electrode rotation speed, ω. The correlation with the collection efficiency is the absolute ratio of ring and disk currents and can be characterized by the following equation [37,40]:

(8) $$N_{k}=-\frac{i_{ring}}{i_{disk}}=N_{geometrical}-\beta^{\frac{2}{3}}\left(1-UA_{1}^{-1}\right)+\frac{1}{2}A_{1}^{-1}A_{2}^{2}\kappa^{2}U\beta^{\frac{4}{3}}-2A_{2}\kappa^{2}T_{2}$$

where *A*_1_ = 1.288, *A*_2_ = 0.643 ν^1/6^ $D_{O_{2}^{•-}}$^1/3^, β = 3ln(*r*_3_/*r*_2_), *U* = *k*^−1^tanh(*A*_1_*k*) and *T*_2_ = 0.718ln(*r*_2_/*r*_1_), whereby *r*_1_–*r*_3_ refer to the radius of the disk and internal and external ring radii, respectively; ν is the kinematic viscosity; ω is the rotation rate; *k* is the rate constant for Equation (2); and $D_{O_{2}^{•-}}$ is the diffusion coefficient of O_2_^•−^. *N_geometrical_* is the geometrical collection efficiency of the RRDE corresponding to the fraction of a species electrochemically generated at the disk. This species is detected at the ring due to the lack of side-reactions with the electrolyte. Equation (8) shows the variation of *N_k_* where the rotation rate and the rate constant (*k*) can be calculated at higher rotation rates, which show that the *N_k_* is close to a constant value. Figure 8a shows the RRDE profiles of the MnO_2_/C sample coating on the disk electrode. The disk and ring currents are recorded in an O_2_-saturated 0.1 M TBAPF_6_/PC solution at rotation rates between 300 and 2100 rpm, with continuous holding of the Pt ring at 2.85 V_Li_. The ring current increases with the rotation rates because the shorter transient time at higher rotation rates reduces the reaction time between O_2_^•−^ and the PC electrolyte so that a higher concentration of superoxide radical can be oxidized at the ring. Also, the *N_k_* increases with rotation rates (ω) and is close to a constant value (0.14) at ω = 2100 rpm, as shown in Figure 8b. The PC-electrolyte decomposition rate constant (*k*) can be calculated by Equation (8) using the *N_k_* value at a rotation speed of 2100 rpm with the kinematic viscosity (ν) and $D_{O_{2}^{•-}}$ listed in Table 1. Table 2 shows the rate constant for producing O_2_^•−^, *k_f_*, and the PC-electrolyte decomposition rate constant, *k*, on the GC and MnO_2_/C-GC electrodes. The value of *k* (1.5 s^−1^) on the GC electrode is close to that of a previously reported measurement (*k* = 1.3 s^−1^) [37]. Obviously, the *k* value on the MnO_2_/C-GC electrode of 2.6 s^−1^ is larger than that on the GC electrode. This result shows that MnO_2_/C is more active for the first step of the ORR (larger rate constant; *k_f_*), producing a higher concentration of O_2_^•−^ and leading to faster PC-electrolyte decomposition due to the attack by a large amount of O_2_^•−^. Therefore, it is important to choose an appropriate electrolyte to avoid decomposition by O_2_^•−^ attack for highly active catalyst applications on the cathode materials in Li-O_2_ batteries. More detailed RRDE experiments and analysis will be carried out to estimate the decomposition rates of various electrolytes with different active catalysts.

**Table 2:** The rate constant for producing O_2_^•−^, *k_f_*, and the PC-electrolyte decomposition rate constant, *k*, on the GC and MnO_2_/C-GC electrodes.

Disk Material/Electrolyte	*k_f_* (cm·s^−1^)	*k* (s^−1^)	Reference
GC/0.1 M TBAPF_6_, PC	1.9 × 10^−2^	1.5	This work
MnO_2_/C-GC/0.1 M TBAPF_6_, PC	4.3 × 10^−2^	2.6	This work
GC/0.2M TBATFSI, PC		1.3	[37]

## 4. Conclusions

A hierarchically mesoporous carbon-supported manganese oxide (MnO_2_/C) has been synthesized by a combination of soft template and hydrothermal methods. SEM and TEM analysis confirmed that hollow MnO_2_ nanorods grow homogeneously on ordered mesoporous carbon frameworks to form a hierarchically mesoporous MnO_2_/C composite structure. The CV and galvanostatic charge–discharge tests indicate that MnO_2_/C composites have excellent catalytic activity towards ORR due to the larger surface area of ordered mesoporous carbon and higher catalytic activity of MnO_2_.

The O_2_ solubility, the diffusion rates of O_2_ and O_2_^•−^ coefficients, the rate constant for producing O_2_^•−^ (*k_f_*), and the PC-electrolyte decomposition rate constant (*k*) of the MnO_2_/C composites have been measured by RRDE experiments and analysis in the 0.1 M TBAPF_6_/PC electrolyte. The results indicate that MnO_2_/C is more active for the first step of the ORR (larger rate constant; *k_f_*), produces a higher concentration of O_2_^•−^, and leads to faster PC-electrolyte decomposition due to the attack by a large amount of O_2_^•−^. The stability of the electrolyte is very important when applying an active catalyst on a cathode material in Li-O_2_ batteries. More detailed RRDE experiments and analysis will be carried out to estimate the decomposition rates of various electrolytes. These results seem to be interesting for the design of advanced Li-O_2_ batteries with high electrochemical performance.
